# Elucidating interfacial charge-transfer dynamics of Ti_3_C_2_T_*x*_ electrodes *via* advanced distribution of relaxation times (DRT) analysis

**DOI:** 10.1039/d5na01183c

**Published:** 2026-04-01

**Authors:** Arya Kannathvalappil, Kaifee Sayeed, Baptiste Py, Sabiar Rahaman, Francesco Ciucci, Kavita Pandey

**Affiliations:** a Centre for Nano and Soft Matter Sciences (CeNS) Shivanapura Bengaluru 562162 India kavitapandey@cens.res.in; b Academy of Scientific and Innovative Research (AcSIR) Ghaziabad 201002 India; c Manipal Academy of Higher Education Manipal Karnataka 576104 India; d Department of Mechanical and Aerospace Engineering, The Hong Kong University of Science and Technology Hong Kong SAR China; e University of Bayreuth, Chair of Electrode Design for Electrochemical Energy Systems Weiherstraße 26 95448 Bayreuth Germany francesco.ciucci@uni-bayreuth.de; f University of Bayreuth, Bavarian Center for Battery Technology (BayBatt), Universitätsstraße 30 95447 Bayreuth Germany

## Abstract

This study employs Distribution of Relaxation Times (DRT) analysis to elucidate the electrochemical charge dynamics within Ti_3_C_2_T_*x*_ MXene-based electrodes. Comprehensive electrochemical characterizations, including cyclic voltammetry (CV) and electrochemical impedance spectroscopy (EIS), were systematically conducted in various electrolytes (H_2_SO_4_, Na_2_SO_4_, and KOH) and on multiple substrates (fluorine-doped tin oxide, carbon paper, and nickel foam) to evaluate charge storage mechanisms. Through DRT analysis, distinct electron-ion transport dynamics and relaxation processes were effectively differentiated, providing deeper insight into electrolyte-substrate interactions. Results demonstrated that acidic electrolytes (H_2_SO_4_) significantly enhanced charge transfer kinetics, while highly porous substrates (nickel foam and carbon paper) improved capacitive behavior. The DRT analysis specifically identified dominant charge-transfer peaks and clarified kinetic limitations, revealing slower dynamics at higher potentials, particularly in Na_2_SO_4_. Overall, this study underscores the capability of DRT analysis to systematically unravel complex electrochemical phenomena, highlighting its importance for optimizing MXene-based energy storage systems.

## Abstract 2

This study employs Distribution of Relaxation Times (DRT) analysis to elucidate the electrochemical charge dynamics within Ti_3_C_2_T_*x*_ MXene-based electrodes.

## Graphical abstract

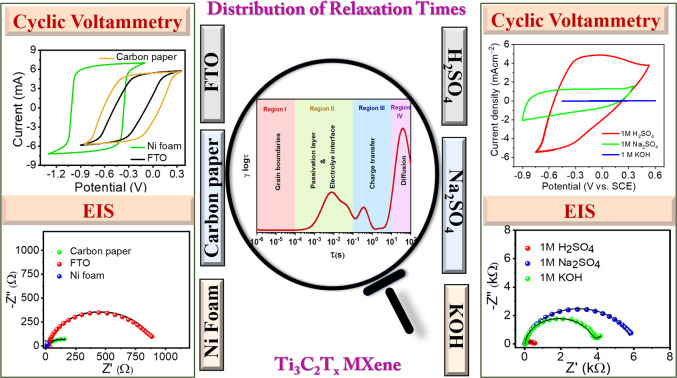

## 1. Introduction

Electrochemical energy storage systems critically rely on accurate characterization techniques to precisely decipher complex charge storage and transfer mechanisms.^1^ Among various analytical methods, Distribution of Relaxation Times (DRT) analysis has emerged as a powerful tool for dissecting impedance data into distinct relaxation processes, facilitating a detailed understanding of electron-ion transport mechanisms and kinetics.^2–4^ Despite its analytical strength, the application of DRT analysis in advanced two-dimensional (2D) materials such as MXenes remains underexplored.

MXenes, a family of two-dimensional transition metal carbides and nitrides, have attracted considerable attention due to their remarkable electrical conductivity, hydrophilicity, tunable surface chemistry, and exceptional capacitive properties.^5–7^ MXenes are typically represented by the chemical formula M_*n*+1_X_*n*_T_*x*_, where M denotes transition metals (*e.g.*, Sc, Ti, Hf, Zr, V, or Nb), X represents carbon and/or nitrogen, and T_*x*_ symbolizes various surface termination groups such as hydroxyl (–OH), oxygen (–O), and fluorine (–F).^8,9^ Specifically, M_2_X structures follow an ABABAB arrangement, while M_3_X_2_ and M_4_X_3_ exhibit an ABCABC stacking sequence. MXene was first discovered in 2011, and various compositions such as Ti_3_C_2_T_*x*_, V_2_CT_*x*_, and Nb_4_C_3_T_*x*_*etc.* have since been explored for energy storage and other applications.^10–12^

Electrolytes are fundamental to determining electrochemical performance, significantly influencing conductivity, ionic mobility, and charge storage mechanisms.^12–14^ Researchers have extensively studied ionic, organic, and aqueous electrolytes. Aqueous electrolytes such as H_2_SO_4_, Na_2_SO_4_, and KOH are especially advantageous due to their high ionic conductivity, environmental friendliness, ease of handling, non-corrosiveness, non-flammability, and relatively low cost compared to organic electrolytes, which typically exhibit flammability, lower conductivity, higher toxicity, and elevated costs.^15^ Previous studies on Ti_3_C_2_T_*x*_-based supercapacitors have demonstrated superior performance in acidic electrolytes; for instance, Lukatskaya *et al.* reported a capacitance of 205 F g^−1^ in 1 M H_2_SO_4_ within a potential window of −0.35 to 0.35 V (*vs.* Ag/AgCl).^16^ Acidic electrolytes typically facilitate proton intercalation, contributing to enhanced pseudo capacitance.^17–21^

Substrate selection further influences electrode performance by affecting interfacial electron-ion dynamics through morphology, conductivity, and porosity variations.^22,23^ Common substrates such as fluorine-doped tin oxide (FTO), carbon paper, and nickel foam differ considerably in these properties, significantly altering the electrochemical response of active materials deposited on them. While numerous studies have investigated MXenes on various substrates, comprehensive investigations covering a broad range of electrolyte and substrate combinations are scarce.^24^ Thus, understanding how the interplay between electrolytes and substrates impacts electrochemical characteristics is essential.

The present study specifically focuses on applying DRT analysis to unravel detailed electrochemical charge dynamics within Ti_3_C_2_T_*x*_ MXene electrodes. Electrochemical behaviors were systematically compared across three distinct aqueous electrolytes: 1 M H_2_SO_4_ (acidic), Na_2_SO_4_ (neutral), and KOH (basic), and evaluated on substrates with diverse morphological and conductive properties (FTO, carbon paper, and nickel foam). Employing cyclic voltammetry (CV), electrochemical impedance spectroscopy (EIS), and particularly emphasizing DRT analysis, this research provides comprehensive insights into how ionic conductivity, electrolyte pH, substrate porosity, and surface morphology influence electron-ion transport dynamics and charge-transfer kinetics. Ultimately, this work highlights the critical role of DRT analysis in deciphering complex electrochemical phenomena, providing essential guidance for optimizing MXene-based energy storage devices.

## 2. Materials and methods

### 2.1. Chemicals and reagents

The Ti_3_AlC_2_ precursor was purchased from Intelligent Materials Private Limited. Analytical grade chemicals, including hydrofluoric acid (HF, 40 wt%), hydrochloric acid (HCl, 12 M), polyvinylidene fluoride (PVDF), *N*-methyl-2-pyrrolidinone (NMP), activated carbon, fluorine-doped tin oxide (FTO), sulfuric acid (H_2_SO_4_), sodium sulfate (Na_2_SO_4_), and potassium hydroxide (KOH), were obtained from Sigma-Aldrich Co., Ltd. Carbon paper and nickel foam were sourced from Synergy Fuel Pvt. Limited. Isopropanol (IPA) was procured from a local supplier and purified before use. Deionized (DI) water (18.2 MΩ cm) used in all experiments was produced using a Sartorius Mini Plus UV water purification system.

### 2.2. Synthesis of Ti_3_C_2_T_*x*_ MXene

Ti_3_C_2_T_*x*_ MXene was synthesized using a selective etching method. Initially, an etchant solution was prepared by combining 12 M HCl, DI water, and 40 wt% HF in a volumetric ratio of 6 : 3 : 1, respectively. Specifically, 6 mL of DI water was placed in a plastic beaker, maintained at 35 °C under constant stirring (400 rpm), followed by the careful addition of 12 mL of 12 M HCl and subsequently 2 mL of 40 wt% HF. After ensuring homogeneous mixing, 1 g of Ti_3_AlC_2_ was gradually added to the etchant and stirred continuously for 36 hours at 35 °C. Following etching, the resulting Ti_3_C_2_T_*x*_ was thoroughly washed *via* repeated centrifugation at approximately 5000 rpm until a neutral pH (∼6) was reached, typically requiring six washing cycles. The product was then dried at 60 °C overnight. For delamination, the washed MXene was dispersed in a 0.5 M LiCl solution (50 mL) and stirred for six hours at room temperature. Subsequently, the suspension underwent multiple centrifugation cycles at 3500 rpm, initially for 5 minutes and then for 1 hour, to remove residual LiCl and impurities thoroughly. Finally, the delaminated Ti_3_C_2_T_*x*_ was vacuum-dried to obtain a pure and exfoliated MXene powder.

### 2.3. Material characterizations

The crystal structure and phase purity of synthesized Ti_3_C_2_T_*x*_ were characterized using X-ray diffraction (XRD) on a SmartLab diffractometer (Rigaku Corp.) employing Cu Kα radiation (*λ* = 0.15418 nm) at 3 kW. Fourier-transform infrared spectroscopy (FTIR) analysis was performed using a PerkinElmer Spectrum 1000 spectrometer. UV-visible absorption spectra were recorded in the wavelength range of 200–800 nm using a Lambda 750 spectrophotometer (PerkinElmer). Raman spectroscopy was conducted using a Horiba Scientific confocal Raman microscope (Xplora Plus V1.2) with a 532 nm laser operated at 25% power.

Surface morphology and elemental analysis were conducted using field emission scanning electron microscopy (FE-SEM, Tescan Mira 3 LMH) coupled with energy dispersive X-ray spectroscopy (EDS, Bruker Quantax 200). High-resolution transmission electron microscopy (HR-TEM, ThermoFisher Talos F200 S) provided detailed microstructural imaging and selected area electron diffraction (SAED) patterns. Atomic force microscopy (AFM, Agilent Technologies) measurements were employed to estimate the thickness and layer count of the synthesized Ti_3_C_2_T_*x*_.

### 2.4. Electrode fabrication and electrochemical measurements

Electrodes were fabricated using a slurry-coating method. The synthesized Ti_3_C_2_T_*x*_ (active material), activated carbon (conducting additive), and PVDF (binder) were mixed in a weight ratio of 8 : 1 : 1. The mixture was mechanically ground in a mortar and pestle for 30 minutes. A controlled quantity of NMP solvent was then added to produce a homogeneous slurry after 12 hours of magnetic stirring.

Three distinct substrates, FTO, carbon paper, and nickel foam, were selected based on their contrasting morphological and conductive characteristics. Before slurry deposition, FTO substrates were cleaned sequentially in ultrasonicated baths of acetone, DI water, and IPA, each lasting 15 minutes. Nickel foam substrates were acid-cleaned in 2 M HCl, rinsed in acetone, and subsequently vacuum-dried at 60 °C for 12 hours. The prepared slurry was uniformly drop-cast onto each substrate, followed by vacuum drying at 80 °C for 24 hours.

Electrochemical characterizations were conducted using a potentiostat/galvanostat (AutoLab PGSTAT302N). Three-electrode setups consisted of Ti_3_C_2_T_*x*_-modified substrates as the working electrode, a platinum mesh as the counter electrode, and either a saturated calomel electrode (for H_2_SO_4_ and Na_2_SO_4_ electrolytes) or Hg|HgO electrode (for KOH electrolyte) as the reference electrode. The electrolytes were freshly prepared 1 M aqueous solutions of H_2_SO_4_, Na_2_SO_4_, and KOH.

CV tests were performed at scan rates ranging from 1 to 150 mV s^−1^ to evaluate electrochemical stability and capacitive behavior. EIS measurements were conducted within a frequency range of 100 mHz to 100 kHz at selected potentials, employing an AC amplitude of 15 mV. The EIS data were analyzed using the Nova 2.1 software, with Kramers–Krönig consistency tests ensuring data reliability. The impedance spectra were further processed through DRT analysis, utilizing zero-mean Gaussian processes with squared-exponential kernels to elucidate electron-ion dynamics precisely.^25–28^

### 2.5. DRT deconvolution

To recover the DRT from the experimental EIS spectra, we approximated the DRT using a finite sum of Gaussian processes with zero mean and squared-exponential kernels.^26^ This probabilistic approach allowed us to estimate the DRT and quantify the uncertainty of the DRT deconvolutions. Another appeal of this approach lies in the optimization of the model parameters by maximizing the experimental evidence. Furthermore, this approach does not rely on a regularization parameter.^25^

## 3. Results and discussions

### 3.1. Powder X-ray diffraction measurements and UV-visible spectroscopy

**Fig. 1:**
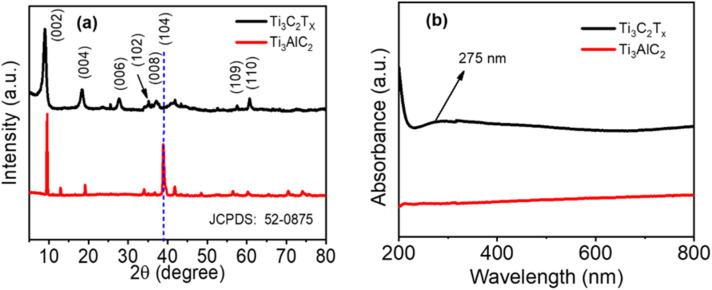
(a) XRD pattern, and (b) UV-visible spectrum of Ti_3_AlC_2_ (untreated) and Ti_3_C_2_T_*x*_.

The XRD patterns of untreated Ti_3_AlC_2_ precursor and synthesized Ti_3_C_2_T_*x*_ are presented in Fig. 1(a). Both patterns correspond closely with the standard diffraction data for Ti_3_AlC_2_ (JCPDS no. 52-0875), confirming the purity of the initial precursor. Upon HF etching and ultrasonication, the characteristic peaks of Ti_3_AlC_2_ within the range of 33° to 43°, particularly the prominent (104) peak at 38.96°, vanished completely, signifying successful removal of aluminum layers and complete transformation into Ti_3_C_2_T_*x*_.^29,30^ Furthermore, a notable shift in the (002) peak from 9.58° for Ti_3_AlC_2_ to 8.92° for Ti_3_C_2_T_*x*_ was observed, attributed to an increased interlayer spacing, as detailed in Table S1 of the SI. The enhanced intensity of the (002) peak additionally validates the successful synthesis of Ti_3_C_2_T_*x*_.^32^

The UV-visible absorbance spectra (Fig. 1(b)) reveal distinct optical characteristics for the precursor and synthesized MXene phases. A prominent absorption peak at approximately 275 nm was observed exclusively for Ti_3_C_2_T_*x*_, absent in the Ti_3_AlC_2_ precursor. This peak, characteristic of localized surface plasmon resonance, underscores the metallic-like electronic behavior of MXene, confirming structural transition and expanded interlayer spacing.^32–34^

### 3.2. FTIR and Raman spectroscopy

**Fig. 2:**
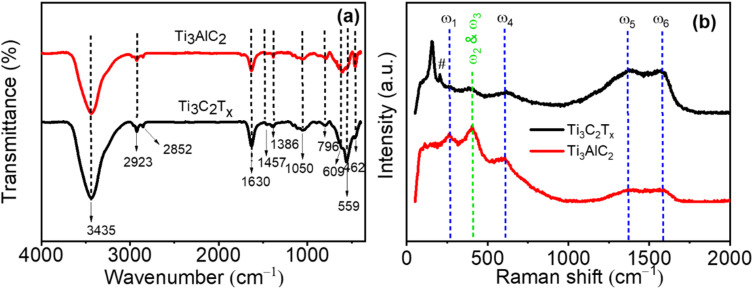
(a) FTIR and (b) Raman spectra of the Ti_3_AlC_2_ phase and the Ti_3_C_2_T_*x*_ MXene.

FTIR spectroscopy was utilized to analyze the composition and surface functionalities of Ti_3_C_2_T_*x*_. Fig. 2(a) presents the comparative FTIR spectra of Ti_3_AlC_2_ and Ti_3_C_2_T_*x*_. The broad absorption peak at approximately 3435 cm^−1^, corresponding to hydroxyl (–OH) stretching vibrations, was notably more intense in Ti_3_C_2_T_*x*_ compared to Ti_3_AlC_2_, reflecting an increase in surface hydroxyl groups resulting from the etching process. The peak at 1630 cm^−1^ corresponds to the bending vibration of the C–OH bond.^34,35^ Additionally, distinct peaks observed at 2923 and 2825 cm^−1^ represent the symmetric and asymmetric stretching vibrations of methyl (–CH_3_) and methylene (–CH_2_) groups, respectively, possibly due to residual amorphous carbon.^34,35^ Peaks at 609 cm^−1^ and 559 cm^−1^ are attributed to Ti–C vibrations and Ti–O deformation vibrations, respectively, while the band at 462 cm^−1^, associated with O–Ti–O vibrations, was more pronounced. Raman spectroscopy further elucidated structural alterations occurring during synthesis (Fig. 2(b)). Prominent peaks *ω*1 (∼267 cm^−1^), *ω*2 (∼409 cm^−1^), and *ω*3 (∼613 cm^−1^), initially attributed to Ti and Al atomic vibrations, underwent significant changes post-etching. The disappearance of the *ω*1 peak, specifically associated with Al vibrations, confirmed effective aluminum removal.^36–38^ Intensified peaks *ω*5 (∼1378 cm^−1^) and *ω*6 (∼1583 cm^−1^), which are usually expressed as D and G band peaks, indicate increased exposure of carbon atoms at the MXene surface. The distinct peak at approximately 208 cm^−1^, denoted by #, is ascribed to out-of-plane vibrations involving carbon and titanium atoms.^39^ The prominent peak at ∼154 cm^−1^, induced by laser power during measurement, reflects partial oxidation of Ti_3_C_2_T_*x*_, further verifying successful MXene formation.

These Raman spectral findings align closely with prior literature, supporting the effective synthesis and structural integrity of Ti_3_C_2_T_*x*_.^39–42^

### 3.3. Morphological and structural analysis

**Fig. 3:**
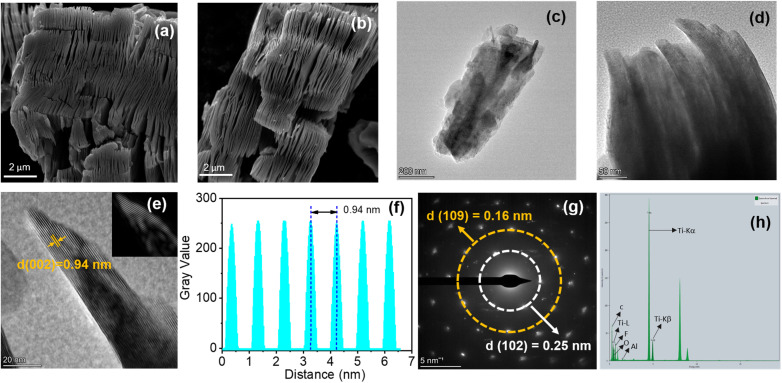
(a and b) FE-SEM, (c and d) Low magnification TEM images, (e) HR-TEM images, (f) plot profile, (g) SAED pattern, and (h) HR-TEM EDS plot of Ti_3_C_2_T_*x*._

Field emission scanning electron microscopy (FE-SEM) and high-resolution transmission electron microscopy (HR-TEM) analyses provided comprehensive morphological insights into the transition from Ti_3_AlC_2_ precursor to Ti_3_C_2_T_*x*_. FE-SEM images depicted the stacked, layered morphology of pristine Ti_3_AlC_2_ (Fig. S1(a and b)). Post-etching, the exfoliated Ti_3_C_2_T_*x*_ displayed a distinctly layered and delaminated structure, clearly illustrating successful aluminum removal (Fig. 3(a and b)). Energy dispersive spectroscopy (EDS) analysis quantitatively validated this transformation, revealing a significant reduction in aluminum content from 18.3% to approximately 0.8%, as summarized in Table S2, confirming efficient etching.

HR-TEM further confirmed the layered structure Fig. 3(c and d) and expanded interplanar spacing of Ti_3_C_2_T_*x*_. Images depicted clear layered stacks with an interlayer spacing of approximately 0.94 nm, consistent with the (002) plane identified in the XRD analysis (Fig. 3(e)). Fig. 3(f) illustrates the plot profile derived from the HR-TEM image, demonstrating uniformity in layer separation. The selected area electron diffraction (SAED) pattern (Fig. 3(g)) showed clear diffraction rings, corroborating the crystalline nature and structural integrity as deduced from XRD patterns.

**Fig. 4:**
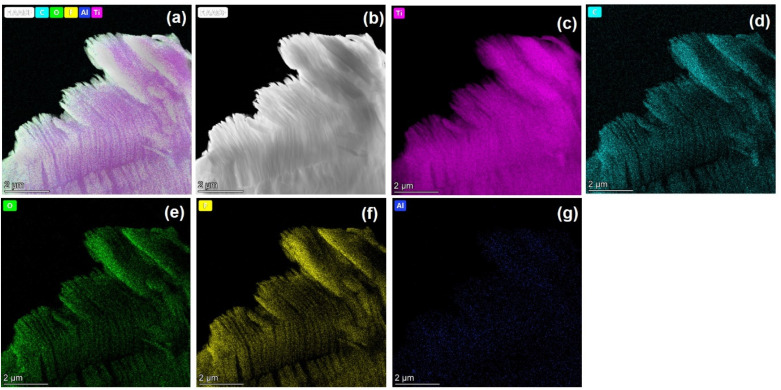
(a) HR-TEM EDS mapping and (b) darkfield image of Ti_3_C_2_T_*x*_, and (c–g) TEM EDS mapping of C, Ti, O, F, and Al.

Elemental mapping using HR-TEM EDS (Fig. 4(a–g)) confirmed homogeneous elemental distribution of carbon (C), titanium (Ti), oxygen (O), and fluorine (F), along with trace residual aluminum (Al), further verifying the efficacy of the etching process. Atomic force microscopy (AFM) analysis (Fig. S2) provided additional structural details, revealing an average layer thickness of approximately 19 nm. Collectively, these rigorous morphological, compositional, and structural characterizations substantiate the successful synthesis and uniform exfoliation of the Ti_3_C_2_T_*x*_ layers.

### 3.4. Electrochemical characterization of Ti_3_C_2_T_*x*_: insights into ionic dynamics and interfacial charge transfer mechanisms

#### 3.4.1. Electrolyte-dependent electrochemical response and charge storage behavior

MXenes, particularly Ti_3_C_2_T_*x*_, exhibit diverse electrochemical responses, including electric double-layer capacitance (EDLC) and pseudo capacitance, significantly influenced by the type of electrolyte and ionic species employed. Literature reports confirm these varied behaviours in salt solutions or ionic liquids, attributing them to the unique two-dimensional layered structure and abundant surface functionalities (–O, –OH, –F) present on MXenes.^43,44^ Such structural characteristics enable rapid intercalation and deintercalation processes of highly mobile ions such as H^+^, Li^+^, Na^+^, and K^+^ across their layers, enhancing charge storage performance.^45,46^ In this study, Ti_3_C_2_T_*x*_ films deposited on fluorine-doped tin oxide (FTO) substrates were analyzed electrochemically in three distinct electrolytes: 1 M H_2_SO_4_, 1 M Na_2_SO_4_, and 1 M KOH. These electrolytes exhibited specific electrochemical stability windows: −0.75 to 0.53 V for H_2_SO_4_, −0.90 to 0.35 V for Na_2_SO_4_, and −0.40 to 0.65 V for KOH. It is worth noting that different synthesis procedures can lead to variations in the surface functional groups of Ti_3_C_2_T_*x*_, as well as electrode fabrication methods can further influence its electrochemical behavior. This study focuses exclusively on Ti_3_C_2_T_*x*_ synthesized using the HF + HCl etching method, and the findings should be interpreted within this context.

##### 3.4.1.1. Cyclic voltammetry studies and ionic mobility analysis

**Fig. 5:**
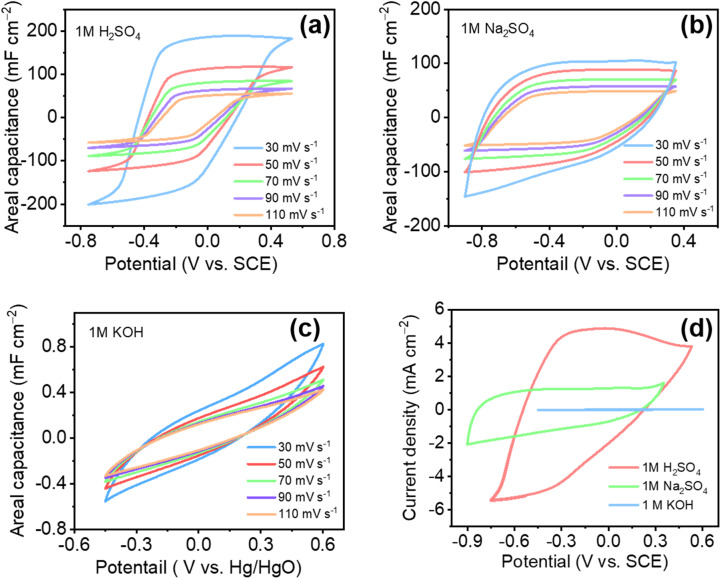
Cyclic voltammetry profiles with varying scan rates for the (a) H_2_SO_4_, (b) Na_2_SO_4_, and (c) KOH electrolytes, and (d) comparison of the CV curves at 10 mV s^−1^ for the three electrolytes.

Fig. 5(a) displays CVs of Ti_3_C_2_T_*x*_ in 1 M H_2_SO_4_ at various scan rates. At lower scan rates (10 mV s^−1^), a prominent broad peak appears around −0.3 V, indicative of pseudocapacitive behavior stemming from redox reactions involving the surface-bound Ti oxidation state transitions.^47^ Conversely, at higher scan rates (110 mV s^−1^), the CV curves adopt a rectangular shape characteristic of EDLC behavior. This transition occurs because the rate of proton diffusion towards the electrode interface exceeds the rate of faradaic reactions, thus promoting capacitive charge storage through ion adsorption/desorption rather than redox processes.^48,49^ The significantly higher current densities observed in acidic media are correlated with ionic mobility. Table 1 summarizes the size, hydrated radius, mobility, and molar conductivity of various ions studied. Notably, H^+^ ions demonstrate the highest ionic mobility (36.20 × 10^−5^ S^−1^ cm^2^ V^−1^), substantially surpassing Na^+^ and K^+^, which results in increased current densities and enhanced electrochemical performance.

**Table 1:** Size, mobility, and conductivity of the different ions considered herein.^50–54^ * and # indicate a measurement at a 1 M and 6 M concentration, respectively

S. no.	Ions	Ionic size (Å)	Hydrated ion size (Å)	Ionic mobility × 10^−5^ (S^−1^ cm^2^ V^−1^)	Molar conductivity (S cm^2^ mol^−1^)
1	H^+^	1.15	2.80	36.20	349.8*
2	Na^+^	0.95	3.58	5.19	50.1*
3	K^+^	1.33	3.31	7.62	73.5^#^
4	OH^−^	1.76	3.00	20.60	198.3^#^
5	SO_4_^2−^	2.90	3.79	8.30	160.0*

Fig. 5(b) illustrates CV profiles of Ti_3_C_2_T_*x*_ in 1 M Na_2_SO_4_, demonstrating predominantly EDLC behavior with nearly ideal rectangular curves across all scan rates (30–110 mV s^−1^). This behavior is consistent with the lower ionic mobility of Na^+^ (5.19 × 10^−5^ S^−1^ cm^2^ V^−1^). Similarly, Ti_3_C_2_T_*x*_ films in 1 M KOH electrolyte (Fig. 5(c)) exhibit comparable EDLC-dominated behaviors but lower current densities (as shown in CV comparison plot Fig. 5(d)) due to the intermediate ionic mobility of K^+^ ions (7.62 × 10^−5^ S^−1^ cm^2^ V^−1^). A direct comparative CV at a scan rate of 10 mV s^−1^ (Fig. 5(d)) clearly reveals the highest capacitance achieved in H_2_SO_4_. This result arises from a combination of proton-driven surface redox reactions and traditional EDLC mechanisms involving protonation of surface oxygen groups and oxidation state changes in Ti.^21,47^

##### 3.4.1.2. Electrochemical impedance spectroscopy (EIS): analysis of interfacial charge transfer kinetics

**Fig. 6:**
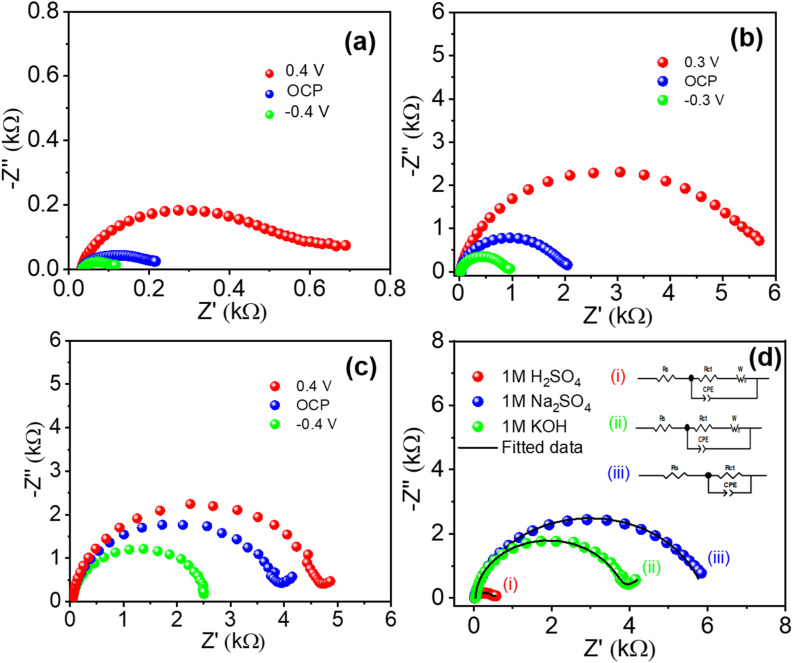
Nyquist plots of the experimental impedances measured below, at, and above the open-circuit voltage for the (a) H_2_SO_4,_ (b) Na_2_SO_4_, and (c) KOH electrolytes, (d) the Nyquist plots of the experimental and fitted impedances at 0.2 V for (i) H_2_SO_4,_ (ii) KOH, and (iii) Na_2_SO_4_.

EIS was employed to investigate the interfacial charge-transfer kinetics and ionic transport characteristics of Ti_3_C_2_T_*x*_ films. Nyquist plots recorded below, at, and above the open-circuit potential (OCP) are shown in Fig. 6(a–c). These plots exhibit semicircles whose diameters correspond to the charge-transfer resistance (*R*_ct_) at the electrode–electrolyte interface.^55,56^ Comparative Nyquist plots and corresponding fitted impedance data at 0.2 V are presented in Fig. 6(d). Detailed fitted parameters, including solution resistance (*R*_s_), charge-transfer resistance, constant phase elements (CPE), and Warburg impedance, are summarized in Table 2. The lowest charge-transfer resistance (*R*_ct_ = 434.64 Ω) is recorded in 1 M H_2_SO_4_ due to its superior proton mobility and conductivity. Significantly higher *R*_ct_ values were obtained for 1 M Na_2_SO_4_ (5908.2 Ω) and 1 M KOH (3657.6 Ω), reflecting their comparatively limited ionic mobility, thereby restricting efficient interfacial ion transfer processes.

**Table 2:** Values of the *R*_s_ and *R*_ct_ resistances, constant phase element (CPE), and Warburg exponent for each electrolyte at 0.2 V

S. no.	Electrolyte	Solution resistance, *R*_s_ (Ω)	Charge transfer resistance, *R*_ct_ (Ω)	CPE		Warburg impedance, *W*
				*Y*_0_ (µMho*S^*n*^)	*n*	
1	1 M H_2_SO_4_	33.8	434.64	65.9	0.79	9.12 mMho*S^0.5^
2	1 M Na_2_SO_4_	20.6	5908.2	27.5	0.89	—
3	1 M KOH	42.3	3657.6	4.89	0.98	1.76 mMho*S^0.5^

##### 3.4.1.3. Bode phase analysis: evaluating capacitive and resistive contributions

The rate-limiting kinetics can be inferred from the phase angle, *Φ*, of the impedance data as a function of the angular frequency *ω*, calculated by the following relation:^57^

1 *Φ* = −tan^−1^ [*Z*′(*ω*)/*Z*′′(*ω*)]

where *Z*′(*ω*) and *Z*′′(*ω*) indicate the real and imaginary parts of the impedance, respectively.

**Fig. 7:**
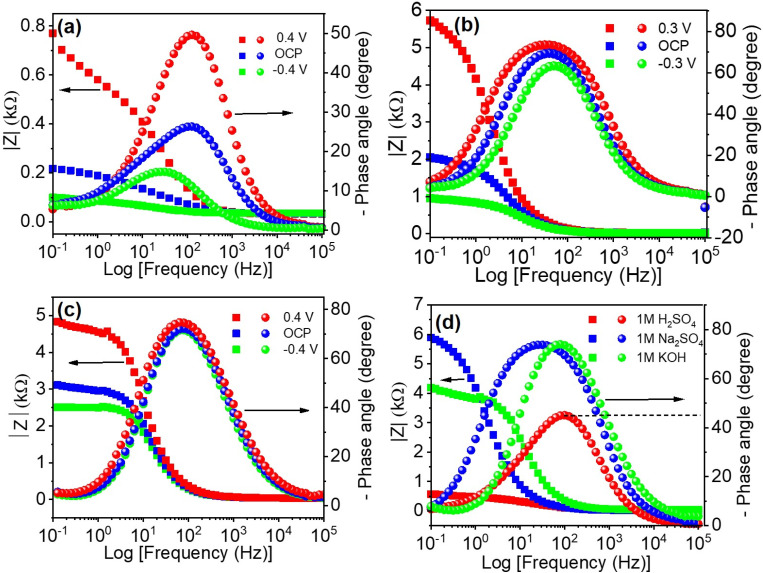
Bode plots of the experimental impedances measured below, at, and above OCP for the (a) H_2_SO_4_, (b) Na_2_SO_4_, and (c) KOH electrolytes, and (d) Bode plots of the experimental impedances for each electrolyte at 0.2 V.

The values of *Φ* are equal to 0°, −90°, and −45° for pure resistive, pure capacitive, and diffusion-limited systems, respectively. Bode phase plots (Fig. 7(a–c)) further elucidate the kinetic behavior of the electrode–electrolyte interfaces. The phase angle (*Φ*) measured from impedance data indicates the relative contributions of capacitive, resistive, and diffusive behaviors at interfaces. For Ti_3_C_2_T_*x*_ in H_2_SO_4_, the phase angle (*Φ* ≈ −45.69°) reveals a mixed capacitive-redox behavior. Conversely, the higher phase angles recorded in Na_2_SO_4_ (−73.36°) and KOH (−74.75°) strongly suggest predominantly capacitive responses. The crossover frequencies (corresponding to *Φ* = −45°) significantly differ across electrolytes, with H_2_SO_4_ showing the lowest frequency (97.23 Hz), indicative of favorable kinetics and rapid interfacial processes.

Post-electrochemical cycling, FE-SEM analysis (Fig. S3) confirmed structural integrity and negligible morphological changes. However, in Na_2_SO_4_ electrolyte, additional EDS analyses (Fig. S4–S6) and elemental mapping (Fig. S7–S9) revealed localized sodium accumulation, suggesting minor electrolyte-induced passivation at the electrode surface.

#### 3.4.2. Electrochemical influence of substrate on Ti_3_C_2_T_*x*_ performance

##### 3.4.2.1. Substrate-dependent cyclic voltammetry studies

**Fig. 8:**
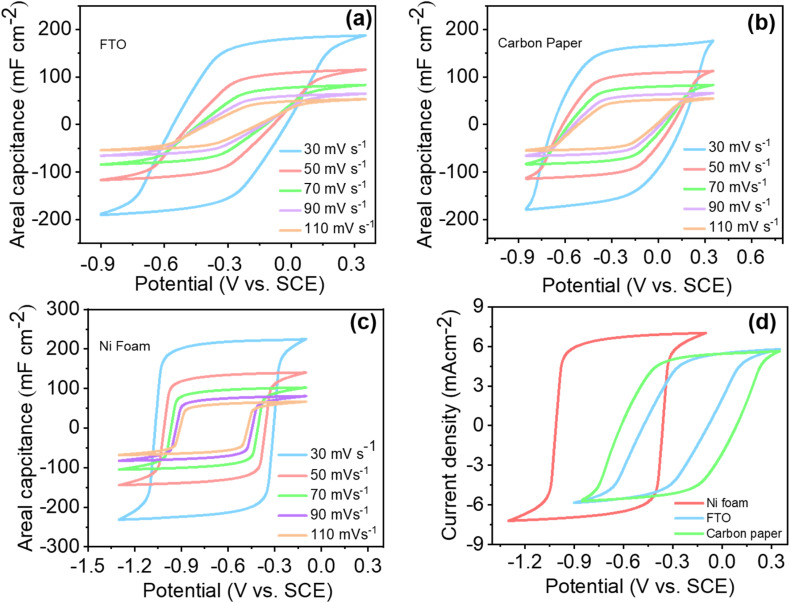
Cyclic voltammetry profiles with varying scan rates for Ti_3_C_2_T_*x*_ on (a) FTO, (b) carbon paper, and (c) nickel foam substrates, and (d) comparison of the CV curves of all three substrates at a scan rate of 50 mV s^−1^.

CV was employed to examine the electrochemical properties of Ti_3_C_2_T_*x*_ deposited on different electrode substrates, specifically FTO, carbon paper, and nickel foam. Fig. 8 illustrates CV analyses performed within distinct potential ranges: −0.9 to 0.35 V for FTO, −0.85 to 0.35 V for carbon paper, and −1.3 to −0.3 V for nickel foam. The total potential windows were approximately comparable across all substrates: 1.25 V for FTO, 1.2 V for carbon paper, and 1.0 V for nickel foam, suggesting minimal substrate influence on the electroactive window.

Fig. 8(a–c) show the CV profiles at varying scan rates from 30 to 110 mV s^−1^ for each substrate. Notably, the areal capacitance progressively reduced with increasing scan rates for all substrates. This behavior is consistent with surface-controlled mechanisms, in which faster potential sweeps reduce the diffusion layer thickness, facilitating greater ion access to electrode surfaces within a shorter time interval. The CV curves for Ti_3_C_2_T_*x*_ on FTO substrates (Fig. 8(a)) exhibit pronounced slanting and blunting characteristics, which intensify with increased scan rates. Fig. 8(d) provides a comparative CV analysis of all three substrates at a fixed scan rate of 50 mV s^−1^. The slanting and blunting effects are attributed primarily to parallel *R*_ct_ and series resistance (*R*_s_). Ti_3_C_2_T_*x*_ on carbon paper exhibits less blunting and slanting, indicative of lower *R*_ct_ compared to FTO substrates. In contrast, Ti_3_C_2_T_*x*_ on nickel foam displays distinctly rectangular CV curves, characteristic of EDLC behavior, correlating directly with its higher capacitance.

##### 3.4.2.2. Quantitative evaluation of substrate-influenced capacitance

The capacitance for Ti_3_C_2_T_*x*_ on each substrate was quantitatively determined using the following equation:^58,59^2
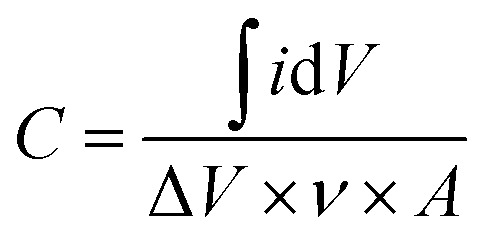
where ∫*i*d*V* is the area inside the cyclic voltammogram, Δ*V* is the potential window, *v* is the scan rate, and *A* is the geometrical surface area of the electrode coated with material and dipped inside the electrolyte.

Capacitance values calculated from CVs at 50 mV s^−1^ are detailed in Table S3. Nickel foam exhibited significantly higher charge storage capabilities due to its enhanced conductivity, increased number of active sites, and porous microstructure. As observed in Fig. 8(d), CV curves for nickel foam were prominently rectangular with vertically oriented characteristics, indicative of substantial capacitive storage. The enhanced performance results from electrolyte penetration into the porous structure, improving ion transport and facilitating more efficient charge transfer. The porosity of substrates follows the order: FTO < carbon paper < nickel foam. Thus, higher porosity directly correlates to improved electrochemical activity.^48^

##### 3.4.2.3. Electrochemical impedance spectroscopy: impact of substrate on charge transfer resistance

**Fig. 9:**
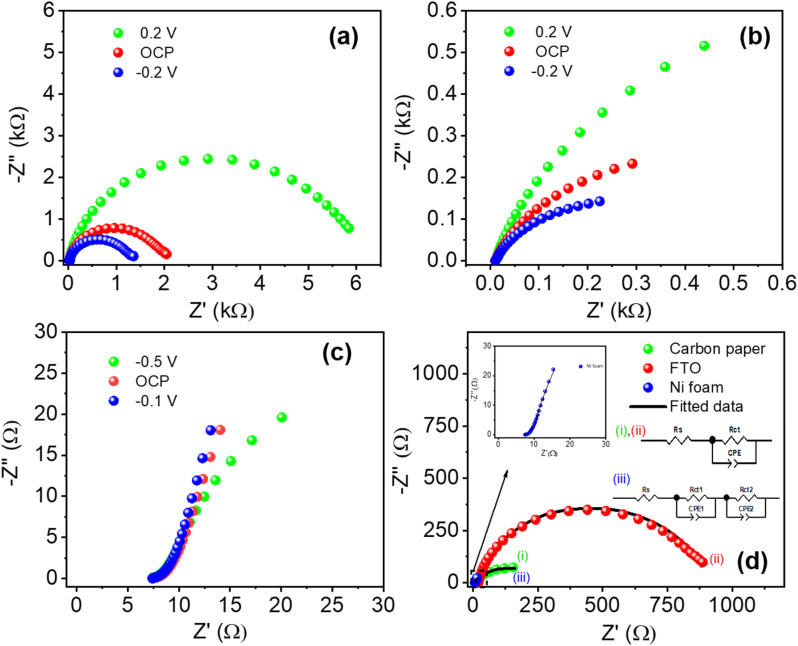
Nyquist plots of the experimental impedances at three different potentials for the (a) FTO, (b) carbon paper, and (c) NF substrates. (d) The Nyquist plots of the experimental and fitted impedances for each substrate at −0.3 V.

EIS was utilized to investigate the interfacial charge transfer dynamics across substrates. Nyquist plots at three distinct potentials for each substrate are displayed in Fig. 9(a–c). Notably, significant differences in *R*_ct_ among substrates were observed, with the lowest *R*_ct_ recorded for nickel foam and the highest for FTO. This trend correlates directly with substrate porosity and conductivity attributes. Nickel foam, characterized by superior conductivity and substantial porosity with pore sizes ranging from 400 nm to 3000 µm, demonstrated the lowest *R*_ct_, indicative of efficient electron transfer kinetics. Carbon paper exhibited intermediate *R*_ct_, whereas FTO presented the highest values.^60^

A detailed comparative analysis of experimental and fitted impedance data at −0.3 V is shown in Fig. 9(d) and summarized in Table 3. Single *R*_ct_/CPE circuit models adequately represented impedance data for carbon paper and FTO. Conversely, nickel foam data required fitting with dual *R*_ct_/CPE elements due to its complex porous structure, which consists of an accessible outer surface and less accessible inner porous regions. These two distinct regions lead to separate charge-transfer resistances. Variations in solution resistance across substrates were also observed, likely due to minor differences in the distances between the working and reference electrodes during electrochemical measurements.

Values of the *R*_s_ and *R*_ct_ resistances and constant phase element (CPE) for each substrate at −0.3 V

**Table:** 

S. no.	Substrate	Solution resistance, *R*_S_ (Ω)	Charge transfer resistance, *R*_ct_ (Ω)	CPE	
				*Y*_0_	*n*
1	FTO	13.8	885	29.8 µMho*S^*n*^	0.854
2	Carbon paper	9.65	244	4.02 mMho*S^*n*^	0.668

**Table:** 

			*R*_ct1_	*R*_ct2_	CPE 1	CPE 2	*n*1	*n*2
3	Nickel foam	7.47	5.37	210	177 mMho*S^*n*1^	68.3 mMho*S^*n*2^	0.292	0.914

##### 3.4.2.4. Bode phase angle analysis: substrate effects on capacitive behavior

**Fig. 10:**
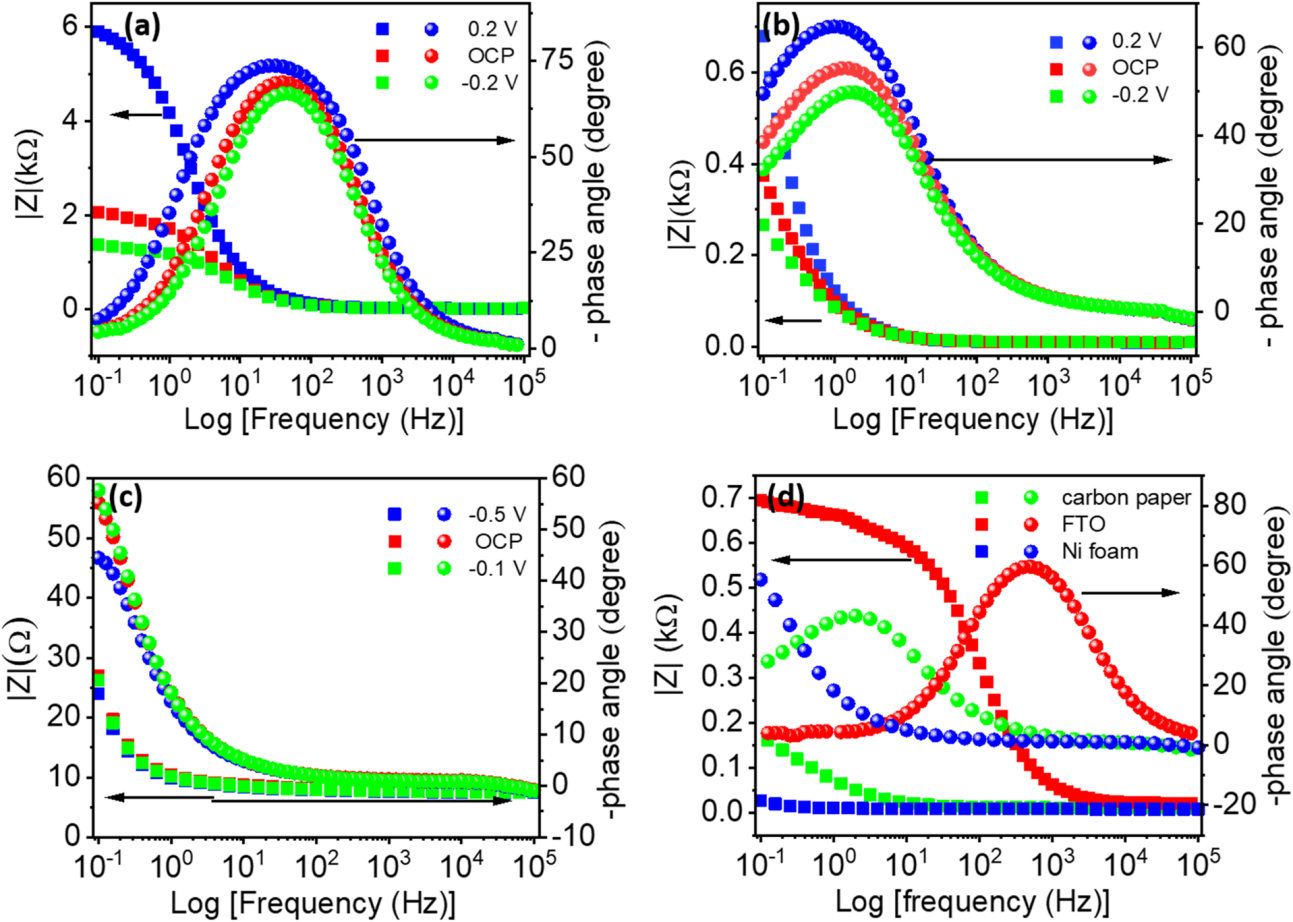
Bode plots of the experimental impedances at three different potentials for the (a) FTO, (b) carbon paper, and (c) nickel foam substrates. (d) The Bode plots of the experimental impedances for each substrate at −0.3 V.

Bode plots depicted in Fig. 10(a–c) further elucidate substrate-dependent capacitive behaviors across different potentials. At OCP, the phase angle (*Φ*) is highest for FTO, indicating superior capacitive behavior relative to the other substrates. A comparative analysis at a constant potential of −0.3 V, illustrated in Fig. 10(d), confirms a descending order of capacitive behavior: FTO (∼70°) > carbon paper (∼60°) > nickel foam (∼5°). FTO demonstrates the greatest capacitive response despite higher resistance, highlighting the substrate-specific trade-offs between capacitive behavior and charge-transfer resistance. To evaluate the practical feasibility and economic viability of the proposed system, a comparative cost analysis of the electrolytes and substrates used in this study was carried out. The per-unit cost of 1 M H_2_SO_4_, 1 M Na_2_SO_4_, and 1 M KOH (calculated per 20 mL), along with the cost per cm^2^ of FTO, carbon paper, and nickel foam, has been systematically analyzed. The detailed cost comparison is provided in Table S4.

This comprehensive evaluation highlights how substrate selection critically influences the electrochemical performance of Ti_3_C_2_T_*x*_ electrodes, emphasizing key substrate characteristics such as porosity, conductivity, and active surface area for optimized energy storage applications.

### 3.5. Distribution of relaxation times (DRT) analysis: unravelling electrochemical processes

#### 3.5.1. Segregation of electrochemical processes *via* DRT methodology

From the EIS spectra, fundamental parameters such as solution resistance, charge-transfer resistance, and diffusion characteristics can be estimated. Nevertheless, accurately distinguishing the various electrochemical processes that occur simultaneously over different timescales using traditional Nyquist impedance spectra remains challenging. To address this limitation, the distribution of relaxation times (DRT) technique has gained prominence as a powerful analytical method, effectively utilized for the detailed study of fuel cells, batteries, solar cells, and electrochemical capacitors.^25–28^ The notable strength of DRT lies in its capability to identify, segregate, and distinctly characterize overlapping physicochemical processes occurring at closely situated timescales.^61–64^

#### 3.5.2. Classification of time-domain regions for detailed analysis

**Fig. 11:**
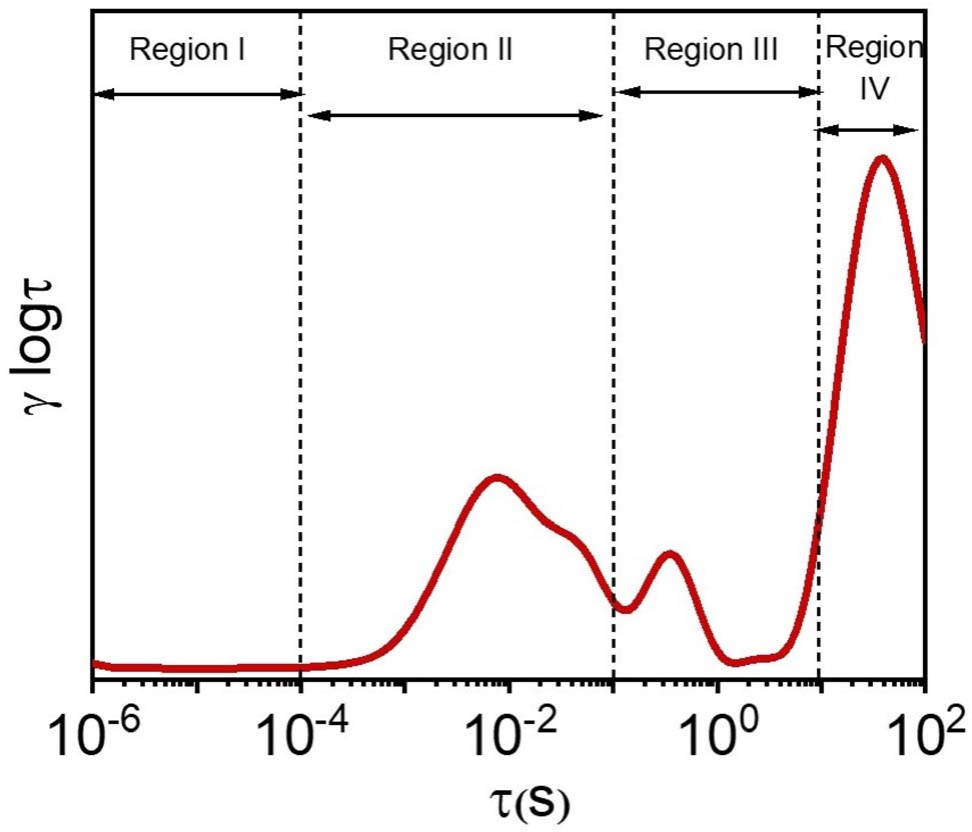
Division of the time domain into different regions.

Prior to converting frequency-domain impedance data to the time-domain representation, the reliability of the collected impedance data was verified using Kramers–Krönig transformations.^65^ Subsequently, the analyzed timescale domain was systematically segmented into four distinct regions, as depicted in Fig. 11. These time-domain regions were categorized as follows: region I (1.0 µs to 0.1 ms), region II (0.1 ms to 0.1 s), region III (0.1 s to 10 s), and region IV (10 s to 1.0 ks). Each region corresponds to distinct electrochemical processes: ionic conductivity and grain boundary phenomena, formation of passivation layers or solid electrolyte interfaces, charge-transfer and electrical double-layer processes, and ionic diffusion processes, respectively.^66–69^ It should be noted that while these assignments are consistent with general DRT interpretation reported in the literature, the precise physicochemical origin of a given time constant can slightly vary depending on the specific materials and electrolyte system.^66–69^

#### 3.5.3. Voltage-dependent DRT spectral behavior across electrolytes

**Fig. 12:**
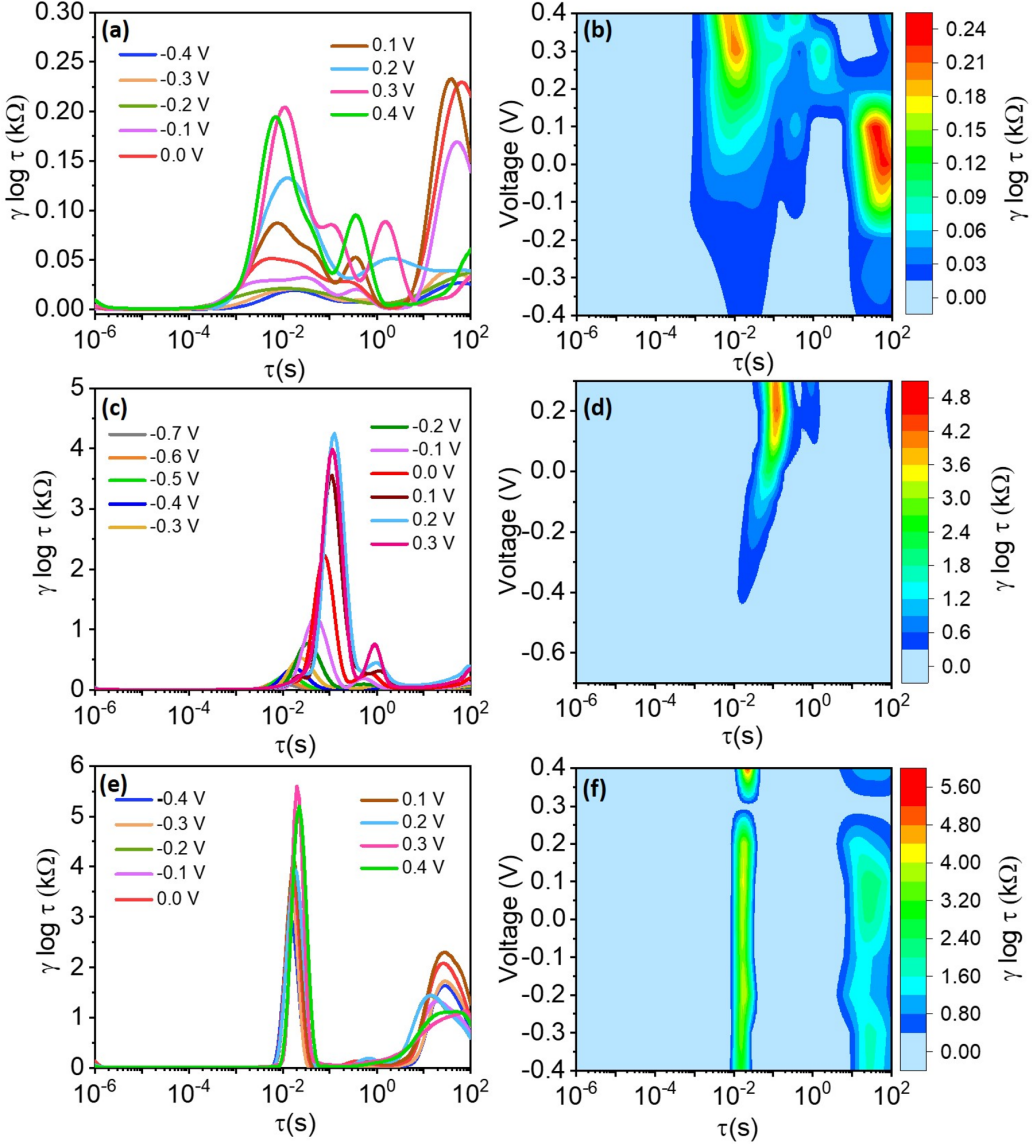
Recovered DRT spectra for (a) H_2_SO_4_, (c) Na_2_SO_4_, and (e) KOH electrolytes at different DC potentials. (b), (d) and (f) corresponding contour plots of the recovered DRTs.

**Fig. 13:**
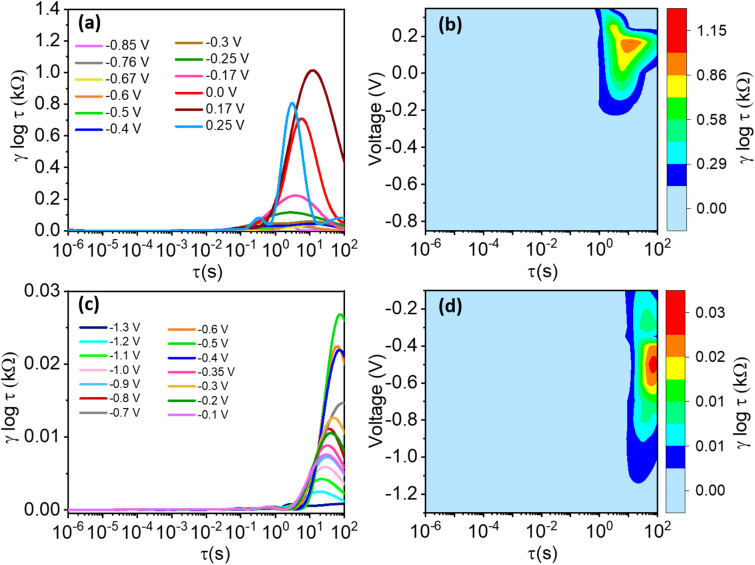
Recovered DRT spectra for (a) carbon paper, and (c) nickel foam substrates at different DC potentials. (b), and (d) corresponding contour plots of the recovered DRTs.

Fig. 12(a, c, e) and 13(a, c) present the recovered DRT spectra as functions of timescale, applied potential, and electrolyte-substrate system composition. The DRT analysis was performed on impedance data collected at the same DC potentials used for EIS, which were chosen within the stable electrochemical window for each system. Complementary contour plots, shown in Fig. 12(b, d, f) and 13(b, d), effectively illustrate how experimental conditions influence DRT profiles. Analysis of the spectra reveals the absence of significant peaks in region I for all tested conditions, while a minor peak in region II was consistently observed, attributed to the formation of passivation layers or solid electrolyte interfaces. Multiple distinguishable peaks appear prominently in region III, indicative of various charge-transfer mechanisms characterized by different relaxation time constants. Specifically, the dominant peak around *τ* ≈ 1.0 s corresponds primarily to ion-electrode charge-transfer interactions. Additional smaller peaks within region III are tentatively associated with ion interactions involving the negligible TiO_2_ layer inherently formed during synthesis on the Ti_3_C_2_T_*x*_ electrode surface.^70^

Considering this study's focus on electrolyte and substrate influences on charge-transfer kinetics, special emphasis was placed on the prominent peak identified in region III. Fig. 12(a, c, e) and 13(a, c) clearly demonstrate the shift of this dominant peak to higher relaxation times with increasing applied potential, signifying progressively sluggish charge-transfer kinetics at elevated potentials. However, this potential-dependent peak shift is less pronounced for H_2_SO_4_ (Fig. 12(a)) and KOH (Fig. 12(e)) compared to Na_2_SO_4_ (Fig. 12(c)). This phenomenon is directly attributed to the significantly higher charge-transfer resistance (*R*_ct_ = 5908.2 Ω) observed in Na_2_SO_4_ (Table 2). Similarly, substrates such as carbon paper and nickel foam displayed more pronounced shifts to higher timescales compared to FTO substrates, where minimal shifts were noted even within the same electrolyte (Na_2_SO_4_). The observed variations in peak intensity align closely with the electrical conductivity properties of these substrates.

To further clarify the influence of the voltage on the recovered DRTs, we also plotted the contour plots of the DRTs in the right panel of Fig. 12 and 13 for the electrolytes and substrates, respectively. For H_2_SO_4_, we notice the presence of two peaks: one peak in the region II that appears as the voltage increases, and one peak in region IV, which becomes predominant around 0 V (Fig. 12(b)). The same trend for the peak in the region II is observed for Na_2_SO_4_, while the second peak does not appear for all applied voltages (Fig. 12(d)). As for KOH, two peaks are observed at approximately the same timescales as for H_2_SO_4_ (Fig. 12(a and f)), with the magnitude of the peak in the region II being twice higher than that in the region IV. Interestingly, the magnitude of these two peaks is approximately voltage-independent, with an exception for a voltage of 0.3 V, at which the peaks appear to vanish (Fig. 12(f)). Regarding the substrates, carbon paper presents a single peak in the region III for voltages comprises between approximately −0.2 and 0.2 V (Fig. 13(b)), whether nickel foam presents a single peak in the region IV, which reached a maximum intensity for a voltage of approximately −0.5 V (Fig. 13(d)).

#### 3.5.4. Polarization resistance (*R*_p_) analysis *via* DRT spectral integration

**Fig. 14:**
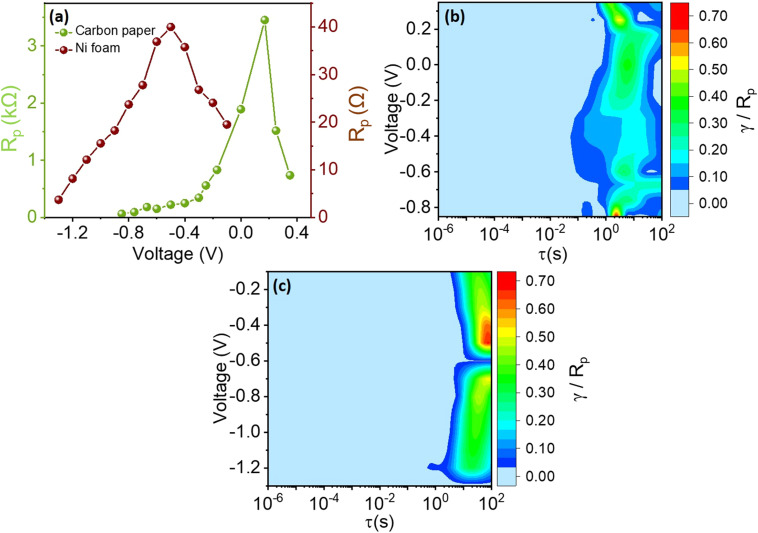
(a) *R*_p_ values as function of the electrolyte and applied voltage. The contour plots of the recovered DRTs for the (b) H_2_SO_4_, (c) KOH, and (d) Na_2_SO_4_ electrolytes are also shown.

**Fig. 15:**
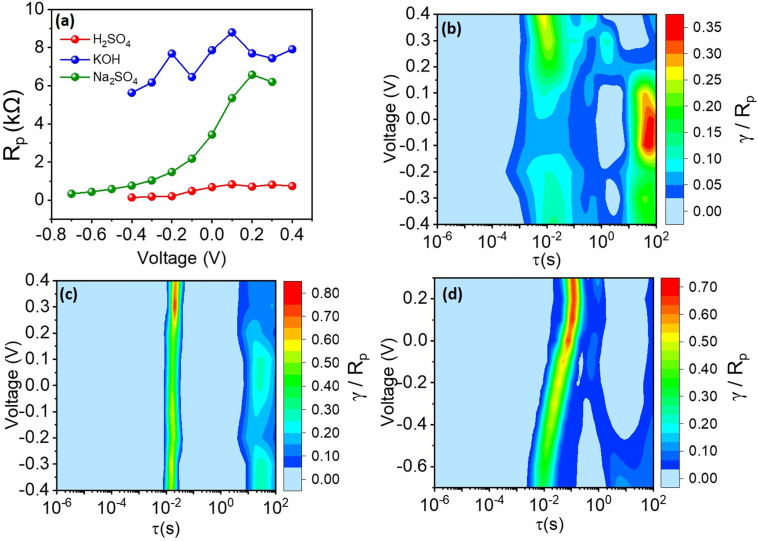
(a) Comparison of *R*_p_ values as function of the substrate and applied voltage. The contour plots of the recovered DRTs for the (b) carbon paper and (c) nickel foam substrates are also shown.

To quantitatively evaluate *R*_p_ the area under the DRT curves was calculated using the trapezoidal rule across different electrolytes and substrates. Fig. 14(a–d) and 15(a–c) illustrate the trends of *R*_p_ with increasing applied potential. A notable increase in *R*_p_ is observed across all electrolyte conditions with increasing potentials, most significantly pronounced in Na_2_SO_4_ electrolyte. Similarly, all substrates demonstrated significant *R*_p_ escalation with potential increments, underscoring a consistent correlation between potential, electrolyte type, substrate material, and resultant polarization resistance.

Through rigorous and systematic DRT analysis, this study effectively delineates complex charge-transfer and diffusion mechanisms, providing comprehensive insight into the influence of electrolyte and substrate selection on electrochemical performance at varying applied potentials.

### 3.6. Integrated mechanistic understanding: correlating interfaces, ionic dynamics, and charge-transfer processes in Ti_3_C_2_T_*x*_ electrodes

The present study systematically correlates findings from electrolyte-dependent electrochemical analyses, substrate-specific investigations, and advanced distribution of relaxation times assessments, establishing a cohesive understanding of ion-electrode interactions and charge-transfer dynamics in Ti_3_C_2_T_*x*_ electrodes. The electrolyte-dependent section distinctly identified the pivotal role of ionic mobility and redox-active interactions, which determine the nature and magnitude of charge storage mechanisms. Specifically, enhanced pseudocapacitance and superior electrochemical performance in acidic electrolytes, notably 1 M H_2_SO_4_, are attributed to high proton mobility and redox-active surface reactions involving Ti oxidation state transitions. Building on these electrolyte-dependent insights, substrate-specific electrochemical characterisation provided complementary understanding by highlighting how substrate morphology, conductivity, and porosity critically influence the electrochemical behavior. Nickel foam's superior performance, characterized by minimal charge-transfer resistance and optimal capacitive behavior, directly results from its advantageous structural and conductive properties, facilitating ion accessibility and efficient electron transport. In contrast, the comparatively higher resistances and altered capacitive characteristics of FTO and carbon paper substrates are consistent with their comparitively lower porosity and conductivity. The subsequent DRT analysis integrates and extends these findings, providing nuanced temporal discrimination of overlapping electrochemical processes occurring at electrode–electrolyte interfaces. The shifts in relaxation times identified through DRT analysis corroborate electrolyte and substrate dependent behaviors, particularly highlighting the slowed kinetics and increased *R*_p_ at elevated potentials. These correlations affirm that electrolyte composition and substrate selection collectively govern the electrochemical interface dynamics and overall device performance. This integrated mechanistic approach successfully bridges electrolyte-dependent, substrate-specific, and temporally resolved analyses, offering comprehensive insights crucial for optimizing Ti_3_C_2_T_*x*_-based electrochemical systems across diverse energy storage and conversion applications.

## 4. Conclusions

Advanced DRT analysis emerged as a powerful tool in this investigation, offering unprecedented insights into the complex interfacial electrochemical dynamics of Ti_3_C_2_T_*x*_ electrodes. Through the DRT technique, distinct electrochemical processes were effectively identified and segregated, notably highlighting the charge-transfer peak around 1.0 s. This peak demonstrated a clear shift towards higher relaxation times with increasing potential, signifying slowed kinetics, particularly pronounced in Na_2_SO_4_ due to its higher *R*_p_. Such behavior was less evident in acidic H_2_SO_4_ and basic KOH electrolytes, reflecting variations in interfacial charge-transfer efficiencies.

Complementing DRT findings, cyclic voltammetry and electrochemical impedance spectroscopy analyses confirmed significant electrolyte dependence in electrochemical responses. Ti_3_C_2_T_*x*_ demonstrated superior electrochemical performance in acidic media (1 M H_2_SO_4_), characterized by the lowest charge-transfer resistance (434.64 Ω), compared to moderate performance in KOH (3657.6 Ω) and relatively poor kinetics in Na_2_SO_4_ (5908.2 Ω). Bode phase analysis further validated these observations, indicating dominant capacitive characteristics in neutral and basic electrolytes (phase angles greater than −70°), contrasted by mixed capacitive-redox behavior in acidic electrolyte (phase angle ∼−45.69°). The influence of substrate material was also critically assessed, revealing that nickel foam substantially enhanced electrochemical performance, achieving the highest areal capacitance (176.29 mF cm^−2^ at 50 mV s^−1^) and exhibiting significantly lower charge-transfer resistance (5.37 Ω). This enhancement is attributed to nickel foam's superior conductivity, porosity, and extensive active surface area, compared to FTO and carbon paper substrates.

This comprehensive study, integrating DRT analysis with electrolyte and substrate-dependent electrochemical characterization, establishes a robust framework for understanding and optimizing Ti_3_C_2_T_*x*_ electrode interfaces. The insights gained here provide critical knowledge for future advancements in MXene-based electrochemical systems for efficient energy storage and conversion applications.

## Conflicts of interest

The authors declare no conflict of interest.

## Supplementary Material

NA-008-D5NA01183C-s001

## Data Availability

The data that support the findings of this study are available from the corresponding author upon reasonable request. The data supporting this article have been included as part of the supplementary information (SI). Supplementary information is available. See DOI: https://doi.org/10.1039/d5na01183c.
