# Supplementary material for: Elucidating interfacial charge-transfer dynamics of Ti3C2Tx electrodes via advanced distribution of relaxation times (DRT) analysis
Source: Nanoscale Adv. 2026 Apr 1;8(9):2942–58. doi: 10.1039/d5na01183c (PMC13058747; doi:10.1039/d5na01183c)
Supplement: NA-008-D5NA01183C-s001 [file NA-008-D5NA01183C-s001.pdf]

Supplementary Information (SI) for Nanoscale Advances.
This journal is © The Royal Society of Chemistry 2026

# Elucidating Interfacial Charge-Transfer Dynamics of $Ti_3C_2T_x$ Electrodes via Advanced Distribution of Relaxation Times (DRT) Analysis

Arya Kannathvalappil[a,b,⊥], Kaifee Sayeed[a,c,⊥], Baptiste Py[d], Sabiar Rahaman[a], Francesco Ciucci[e,f,*], Kavita Pandey[a,b,c,*]

[a] Centre for Nano and Soft Matter Sciences (CeNS), Shivanapura, Bengaluru 562162, India

[b] Academy of Scientific and Innovative Research (AcSIR), Ghaziabad 201002, India

[c] Manipal Academy of Higher Education, Manipal, Karnataka, 576104, India

[d] Department of Mechanical and Aerospace Engineering, The Hong Kong University of Science and Technology, Hong Kong SAR, China

[e] University of Bayreuth, Chair of Electrode Design for Electrochemical Energy Systems, Weiherstraße 26, 95448 Bayreuth, Germany

[f] University of Bayreuth, Bavarian Center for Battery Technology (BayBatt), Universitätsstraße 30, 95447 Bayreuth, Germany

**Table S1.** The inter-planar separation (d-spacing ) of $Ti_3AlC_2$ MAX phase and $Ti_3C_2T_x$ MXene calculated with Bragg's law.

| **$Ti_3AlC_2$ (MAX Phase)** | | **$Ti_3C_2T_x$ (MXene)** | |
|---|---|---|---|
| **2θ** | **d- Spacing** | **2θ** | **d- Spacing** |
| 9.57706 | 9.227542 | 8.92692 | 9.898064 |
| 12.80438 | 6.908092 | 18.33966 | 4.833668 |
| 19.17237 | 4.625565 | 23.00505 | 3.862874 |
| 33.98769 | 2.635586 | 25.56701 | 3.481303 |
| 34.98404 | 2.562772 | 27.74141 | 3.213176 |
| 35.98039 | 2.494057 | 35.10773 | 2.554026 |
| 36.73849 | 2.444313 | 37.22321 | 2.413589 |
| 38.96945 | 2.309361 | 41.15307 | 2.191725 |
| 41.98016 | 2.150437 | 41.5186 | 2.17327 |
| 43.21477 | 2.091817 | 42.74418 | 2.113751 |
| 45.20748 | 2.004134 | 43.34321 | 2.085916 |
| 48.56475 | 1.873143 | 52.49351 | 1.741823 |
| 52.94004 | 1.728178 | 57.55191 | 1.600172 |
| 56.55723 | 1.62593 | 62.05853 | 1.494347 |
| 60.28272 | 1.534049 | 65.61571 | 1.421681 |
| 65.5244 | 1.423441 | 75.11873 | 1.263651 |
| 70.63611 | 1.332434 | | |
| 73.99323 | 1.280062 | | |

**Table S2.** EDS data of aluminum etching obtained from FESEM EDS.

| Condition | Atomic wt. % of Aluminium |
|---|---|
| Before etching | 18.29% |
| After etching | 0.83% |

**Table S3**. Capacitance values calculated from the CV curve at 50 $mVs^{-1}$ for each electrolyte and each substrate given an effective area of the electrode of $1cm^{-2}$.

| S. No. | Electrolyte/Substrate | Capacitance ($mF/cm^2$) |
|---|---|---|
| 1. | $H_2SO_4$ | 89.36 |
| 2. | $Na_2SO_4$ | 126.67 |
| 3. | KOH | 0.24 |
| 4. | FTO | 68.95 |
| 5. | Carbon Paper | 119.48 |
| 6. | Nickel Foam | 176.29 |

**Table S4.** Comparative analysis of the per-unit cost of electrolytes (1 M $H_2SO_4$, 1 M $Na_2SO_4$, and 1 M KOH; calculated per 20 mL) and substrates (FTO, carbon paper, and nickel foam; calculated per $cm^2$) used in this study.

| S. No. | Electrolyte/ Substrate | Source | Approximate Cost (in INR) |
|---|---|---|---|
| 1. | 1M $H_2SO_4$ | Merck | 78.8 per 20 ml |
| 2. | 1M $Na_2SO_4$ | Merck | 29.8 per 20 ml |
| 3. | 1M KOH | Merck | 6.9 per 20 ml |
| 4. | FTO | Sigma Aldrich | 9.4 per $cm^2$ |
| 5. | Carbon paper | Sainergy Fuel Cell India Private Limited | 13.6 per $cm^2$ |
| 6. | Nickel Foam | Sigma Aldrich | 20.0 per $cm^2$ |

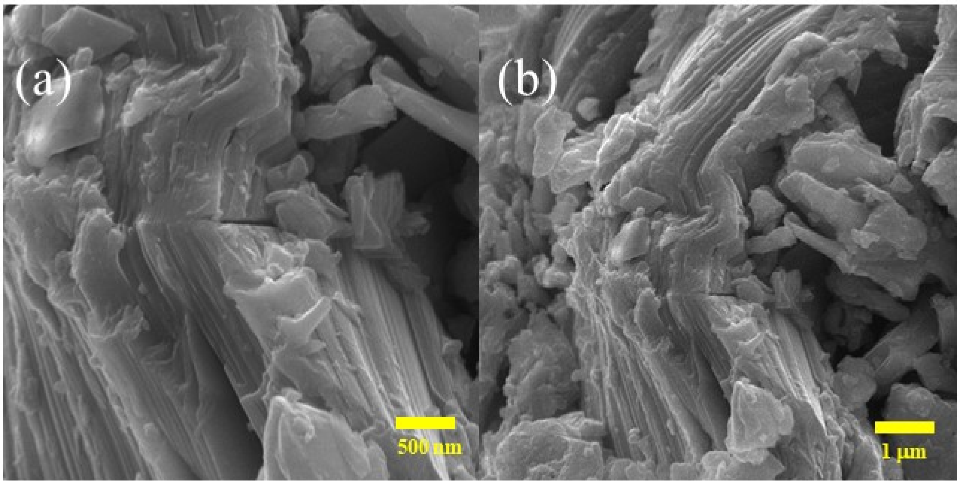


**Figure S1.**(a-b) FESEM image of the $Ti_3AlC_2$ MAX phase at different magnifications.

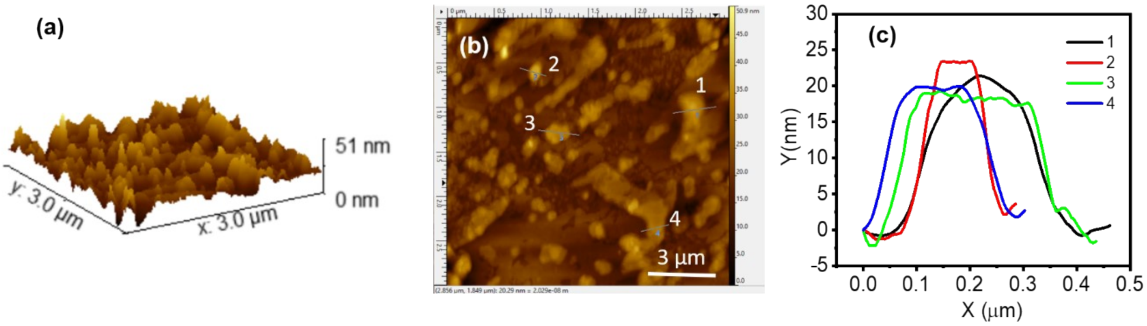


**Figure S2.** Atomic force microscopy images of $Ti_3C_2T_x$ (a) Three-dimensional image, (b) showing four arbitrarily different selected regions for analysis, and (c) height profile corresponding to four different regions.

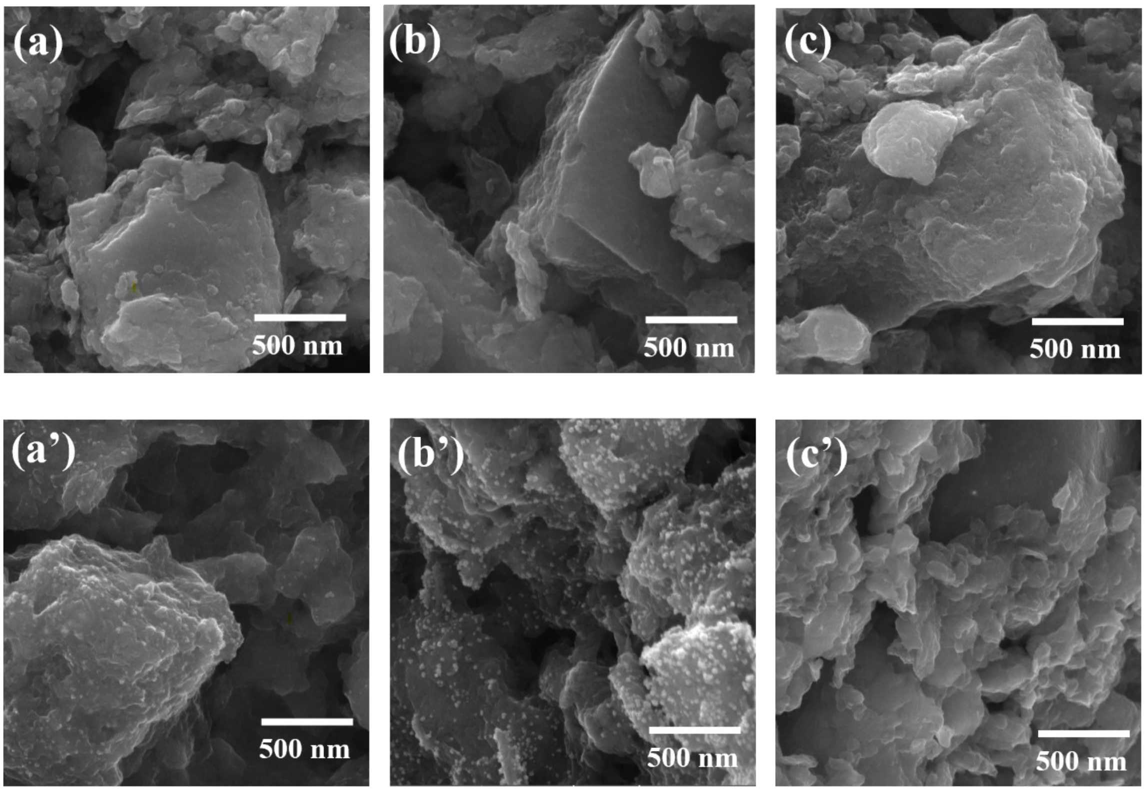


**Figure S3.** FESEM image of the electrode before and after using in (a and a') $H_2SO_4$, (b and b') $Na_2SO_4$, and (c and c') KOH.

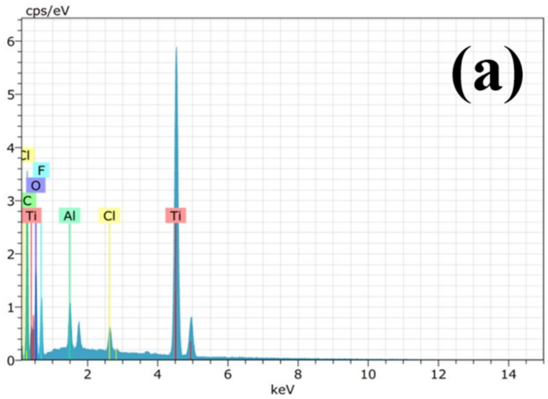


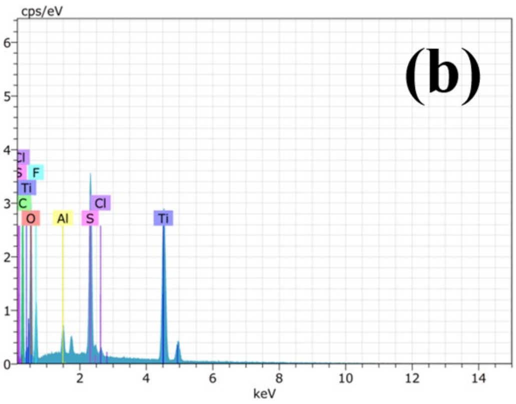


| Element | Series | unn. C [wt.%] | norm. C [wt.%] | Atom. C [at.%] | Error (3 Sigma) [wt.%] |
|---|---|---|---|---|---|
| Titanium | K-series | 46.72 | 42.90 | 18.91 | 4.14 |
| Carbon | K-series | 24.48 | 22.48 | 39.49 | 9.25 |
| Oxygen | K-series | 22.35 | 20.53 | 27.07 | 9.12 |
| Fluorine | K-series | 12.34 | 11.33 | 12.59 | 5.27 |
| Chlorine | K-series | 1.25 | 1.15 | 0.68 | 0.21 |
| Aluminium | K-series | 1.76 | 1.62 | 1.27 | 0.33 |
| | Total: | 108.90 | 100.00 | 100.00 | |

| Element | Series | unn. C [wt.%] | norm. C [wt.%] | Atom. C [at.%] | Error (3 Sigma) [wt.%] |
|---|---|---|---|---|---|
| Oxygen | K-series | 30.05 | 29.57 | 33.31 | 11.43 |
| Carbon | K-series | 29.70 | 29.23 | 43.86 | 11.30 |
| Titanium | K-series | 22.98 | 22.62 | 8.52 | 2.09 |
| Fluorine | K-series | 9.98 | 9.82 | 9.32 | 4.32 |
| Sulfur | K-series | 7.54 | 7.42 | 4.17 | 0.88 |
| Chlorine | K-series | 0.41 | 0.40 | 0.21 | 0.13 |
| Aluminium | K-series | 0.94 | 0.92 | 0.62 | 0.22 |
| Hydrogen | K-series | 0.00 | 0.00 | 0.00 | 0.00 |
| | Total: | 101.60 | 100.00 | 100.00 | |

**Figure S4.** EDS of the electrode before (a) and after (b) using the $H_2SO_4$ electrolyte.

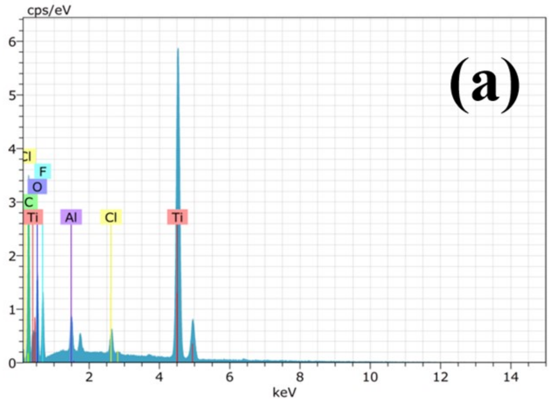


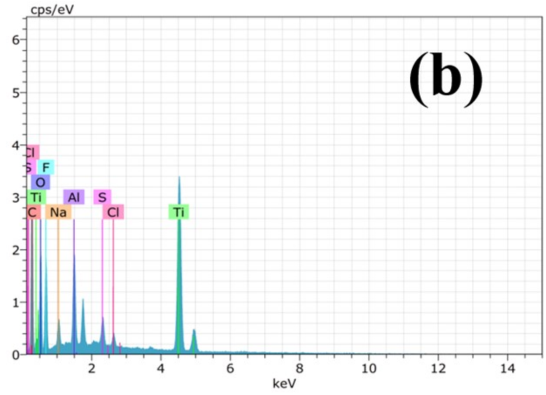


| Element | Series | unn. C [wt.%] | norm. C [wt.%] | Atom. C [at.%] | Error (3 Sigma) [wt.%] |
|---|---|---|---|---|---|
| Titanium | K-series | 45.07 | 43.19 | 19.15 | 3.99 |
| Carbon | K-series | 22.73 | 21.78 | 38.49 | 8.60 |
| Oxygen | K-series | 21.31 | 20.43 | 27.10 | 8.67 |
| Fluorine | K-series | 12.62 | 12.09 | 13.51 | 5.31 |
| Chlorine | K-series | 1.22 | 1.17 | 0.70 | 0.21 |
| Aluminium | K-series | 1.39 | 1.34 | 1.05 | 0.28 |
| | Total: | 104.35 | 100.00 | 100.00 | |

| Element | Series | unn. C [wt.%] | norm. C [wt.%] | Atom. C [at.%] | Error (3 Sigma) [wt.%] |
|---|---|---|---|---|---|
| Carbon | K-series | 27.12 | 27.98 | 43.73 | 10.18 |
| Titanium | K-series | 27.83 | 28.71 | 11.26 | 2.51 |
| Oxygen | K-series | 21.07 | 21.75 | 25.52 | 8.39 |
| Fluorine | K-series | 15.15 | 15.63 | 15.45 | 6.10 |
| Sodium | K-series | 1.17 | 1.21 | 0.99 | 0.31 |
| Aluminium | K-series | 2.87 | 2.96 | 2.06 | 0.48 |
| Chlorine | K-series | 0.58 | 0.60 | 0.32 | 0.14 |
| Sulfur | K-series | 1.13 | 1.16 | 0.68 | 0.21 |
| | Total: | 96.91 | 100.00 | 100.00 | |

**Figure S5.** EDS of the electrode before (a) and after (b) using the $Na_2SO_4$ electrolyte.

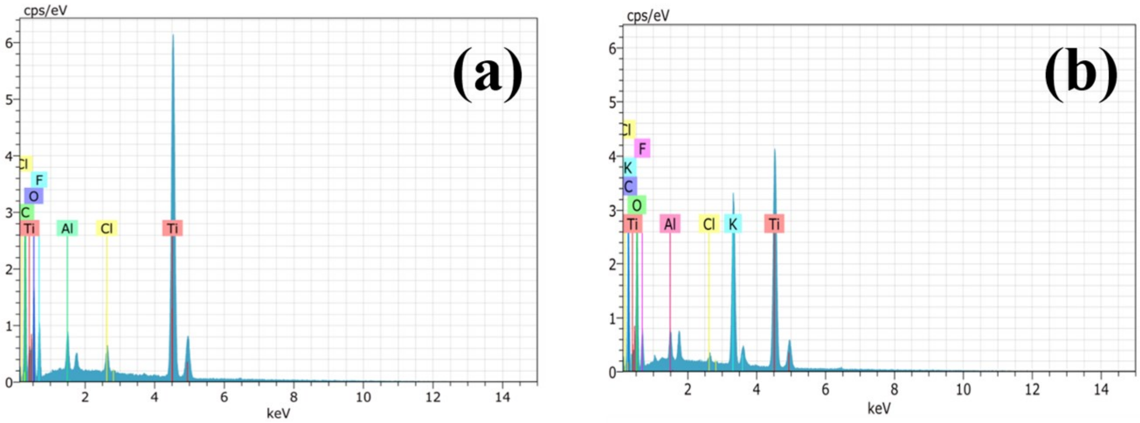


(a)

| Element | Series | unn. C [wt.%] | norm. C [wt.%] | Atom. C [at.%] | Error (3 Sigma) [wt.%] |
|---|---|---|---|---|---|
| Titanium | K-series | 48.80 | 45.80 | 20.76 | 4.32 |
| Carbon | K-series | 22.13 | 20.77 | 37.53 | 8.43 |
| Oxygen | K-series | 22.55 | 21.16 | 28.71 | 9.19 |
| Fluorine | K-series | 10.55 | 9.90 | 11.31 | 4.62 |
| Chlorine | K-series | 1.20 | 1.12 | 0.69 | 0.21 |
| Aluminium | K-series | 1.33 | 1.25 | 1.00 | 0.27 |
| | Total: | 106.55 | 100.00 | 100.00 | |

(b)

| Element | Series | unn. C [wt.%] | norm. C [wt.%] | Atom. C [at.%] | Error (3 Sigma) [wt.%] |
|---|---|---|---|---|---|
| Titanium | K-series | 33.95 | 32.82 | 14.65 | 3.04 |
| Oxygen | K-series | 33.49 | 32.38 | 43.26 | 12.75 |
| Carbon | K-series | 15.61 | 15.09 | 26.86 | 6.09 |
| Potassium | K-series | 11.45 | 11.07 | 6.05 | 1.14 |
| Fluorine | K-series | 7.42 | 7.18 | 8.08 | 3.45 |
| Aluminium | K-series | 1.14 | 1.11 | 0.88 | 0.25 |
| Chlorine | K-series | 0.37 | 0.36 | 0.22 | 0.12 |
| | Total: | 103.45 | 100.00 | 100.00 | |

**Figure S6.** EDS of the electrode before (a) and after (b) using the KOH electrolyte

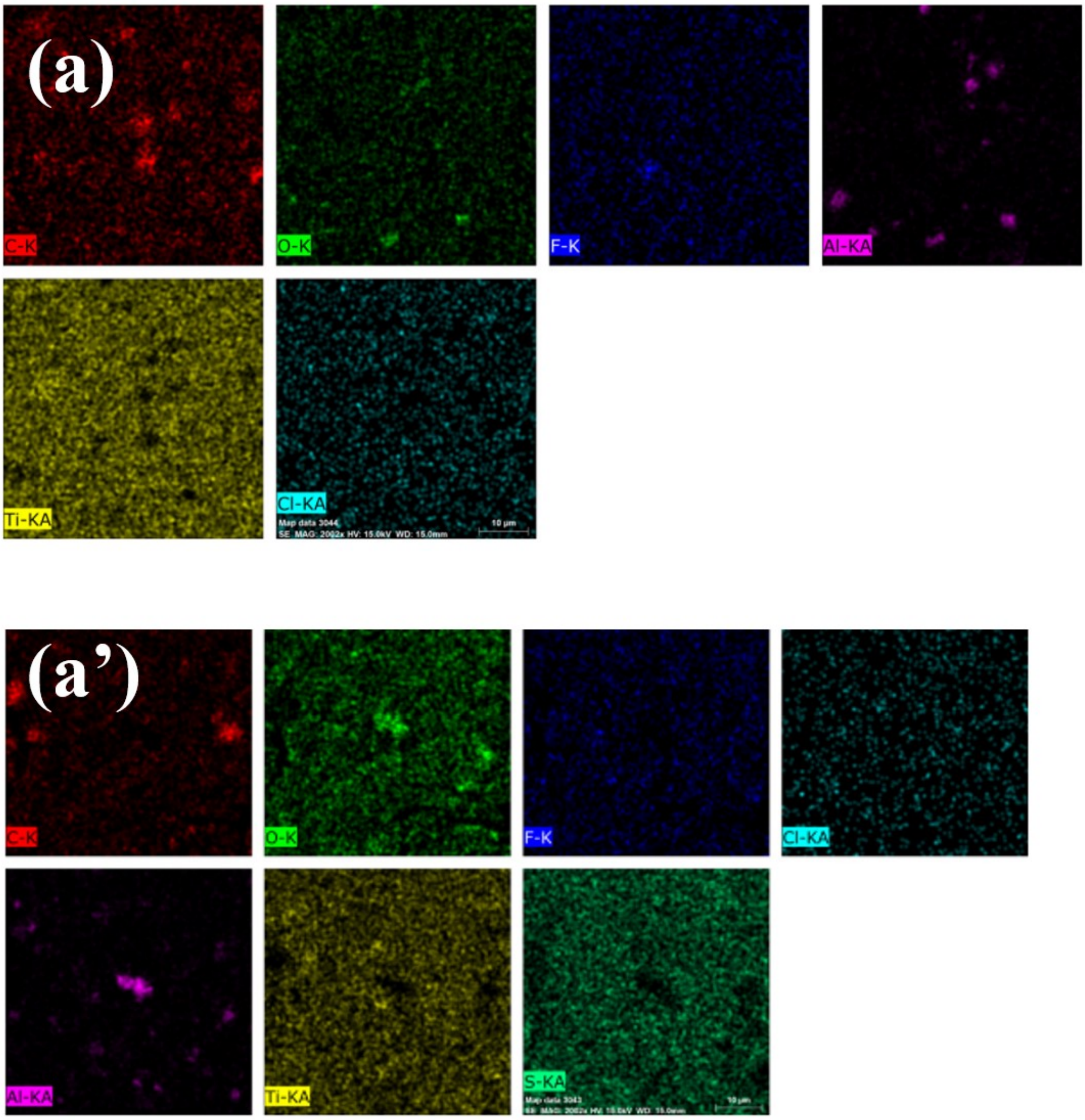


.

**Figure S7.** Mapping of the electrode before (a) and after (a') electrochemical experiments in $H_2SO_4$ electrolyte.

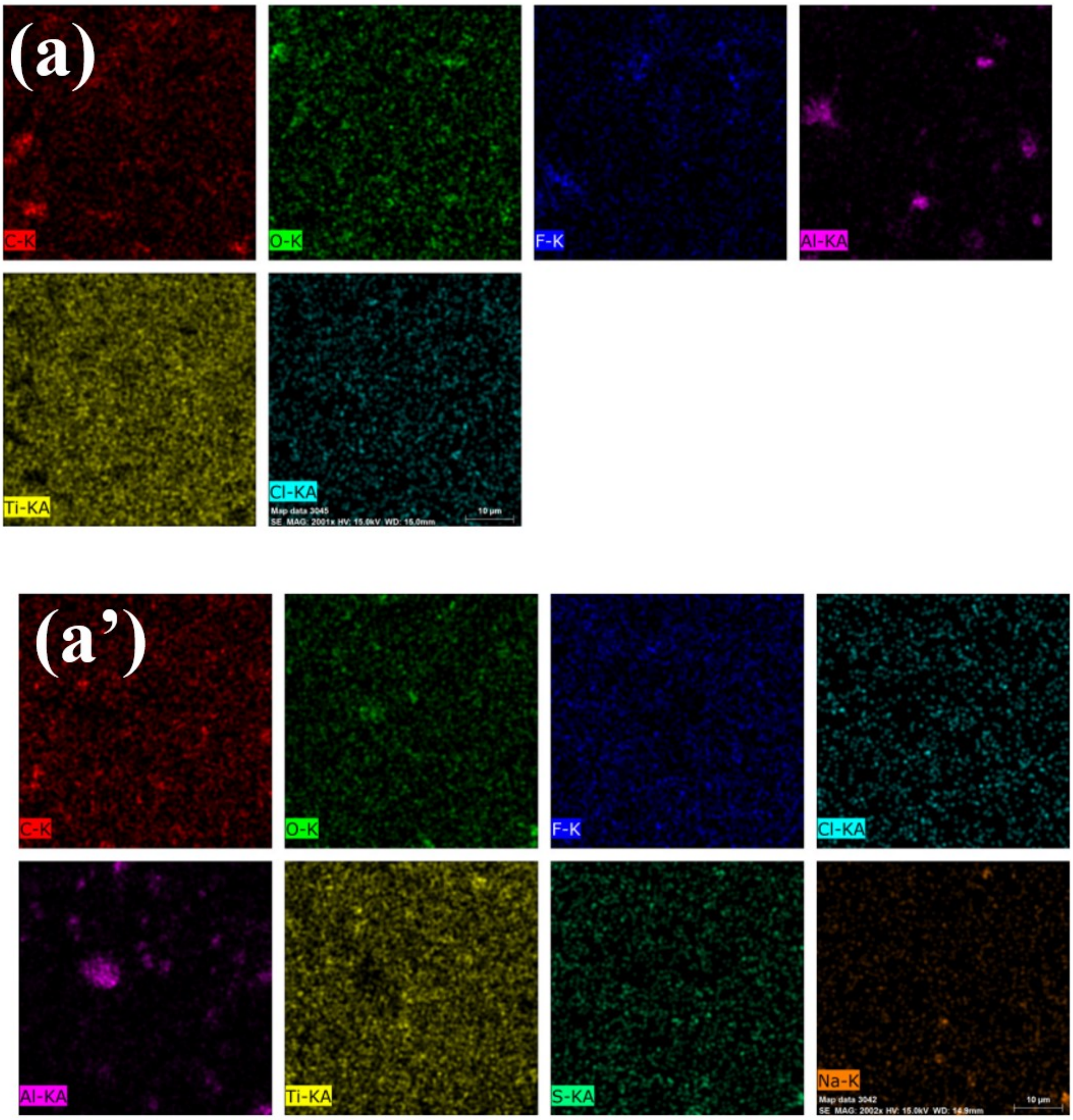


**Figure S8.** Mapping of the electrode before (a) and after (a’) electrochemical experiments in $Na_2SO_4$ electrolyte.

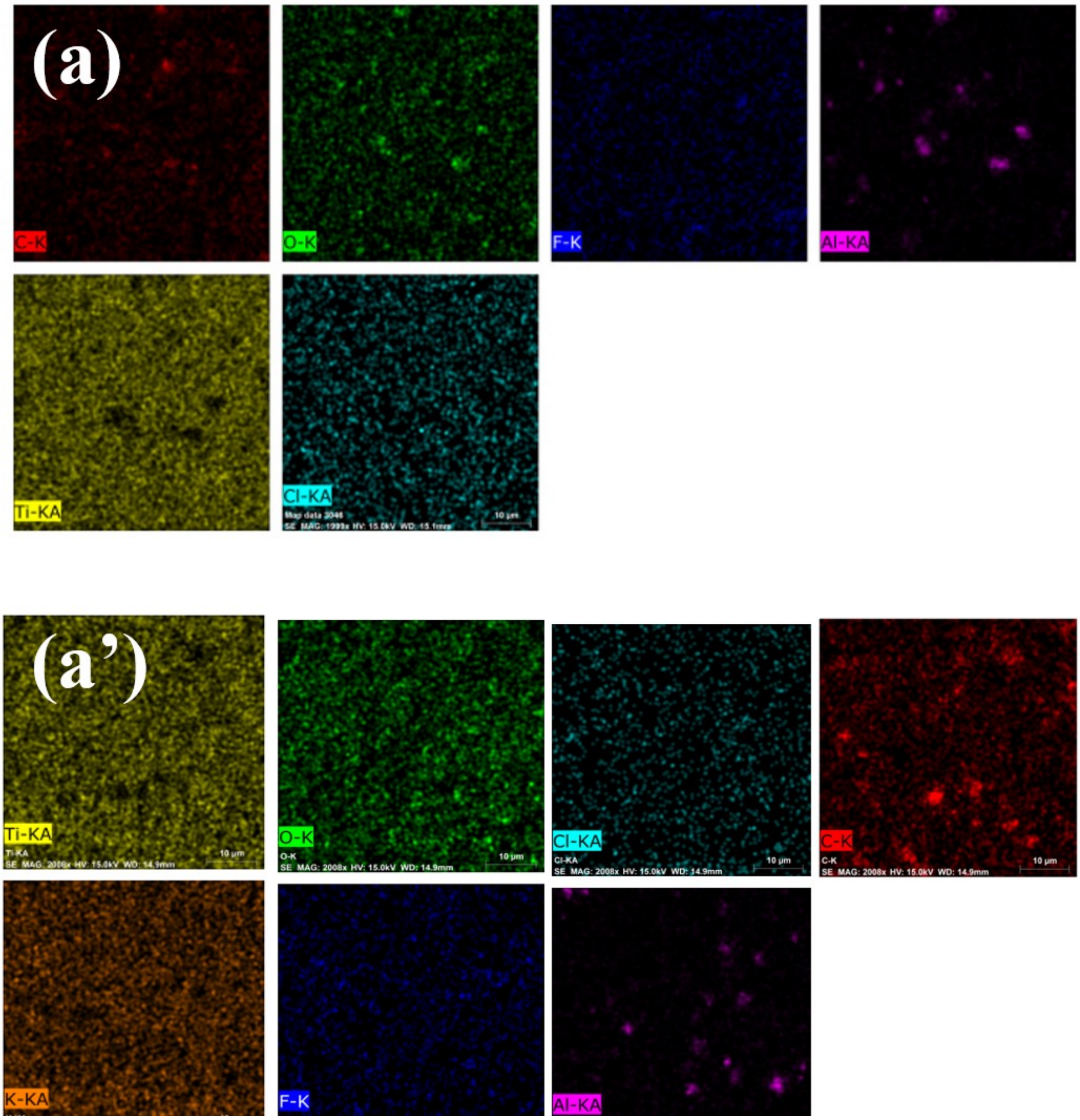


**Figure S9.** Mapping of the electrode before (a) and after (a') electrochemical experiments in KOH electrolyte.
